# The role of tunneling nanotubes during early stages of HIV infection and reactivation: implications in HIV cure

**DOI:** 10.1515/nipt-2022-0015

**Published:** 2023-01-04

**Authors:** Silvana Valdebenito, Akira Ono, Libin Rong, Eliseo A. Eugenin

**Affiliations:** Department of Neurobiology, University of Texas Medical Branch (UTMB), Galveston, TX, USA; Department of Microbiology & Immunology, University of Michigan Medical School, Ann Arbor, MI, USA; Department of Mathematics, University of Florida, Gainesville, FL, USA

**Keywords:** gap junctions, HIV, reactivation, reservoirs, tunneling nanotubes, virological synapses

## Abstract

Tunneling nanotubes (TNTs), also called cytonemes or tumor microtubes, correspond to cellular processes that enable long-range communication. TNTs are plasma membrane extensions that form tubular processes that connect the cytoplasm of two or more cells. TNTs are mostly expressed during the early stages of development and poorly expressed in adulthood. However, in disease conditions such as stroke, cancer, and viral infections such as HIV, TNTs proliferate, but their role is poorly understood. TNTs function has been associated with signaling coordination, organelle sharing, and the transfer of infectious agents such as HIV. Here, we describe the critical role and function of TNTs during HIV infection and reactivation, as well as the use of TNTs for cure strategies.

## Introduction

Since the beginning of the human immunodeficiency virus-1 (HIV) pandemic in 1981 [1], HIV has infected 85 million people worldwide, and currently, 38.5 million individuals live with the virus [2–4]. The introduction of the antiretroviral therapy (ART) in 1987 to control viral replication has transformed HIV from a deadly to a chronic disease; however, a cure is still needed [5]. The major difficulty in curing HIV is the early generation of long-lasting latently infected viral reservoirs that hide from the immune system and avoiding ART’s toxic effects [6–9]. Long-term ART has decreased the pool of active and latently infected cells resulting in minimal to undetectable systemic viral replication in most treated individuals [6], [7], [8, 10], but upon ART interruption, a rapid systemic viral rebound occurs [9, 11], [12], [13], [14]. However, the mechanisms of viral reactivation and spread to repopulate the body from the few remaining viral reservoirs are not well known and are the focus of the current manuscript.

In the early stages of HIV infection, there are several anatomic routes for HIV transmission, including the vagina, anus, rectum, and penis/foreskin. These tissues have efficient physical (epithelium and mucus) and biological barriers (T-cells, macrophages, and antimicrobial factors) that prevent viral and bacterial infections [15–18]. However, infectious virions or HIV-infected cells cross into the uninfected host if these barriers fail. It has been calculated that HIV transmission occurs in 0.1% of unprotected vaginal and 1.4% of anal intercourse [19]. Several groups have shown that these infectious agents can move up to 10 µm into the host tissue and establish contact with T-cells, dendritic cells, or macrophages to transfer the infection into the new host [20, 21]. Furthermore, has been calculated that the half-life of the virion in solution is around 3–4 min [22], and HIV-infected cells can survive up to 1.39 days in tissues [23, 24]. Experiments in macaques injected with purified viruses confirmed that the virion half-life is 3–4 min in serum [25, 26]. It has been estimated that a single HIV-infected cell could produce between 10^3^–10^4^ viral particles before undergoing apoptosis [22, 24]. However, most of the virus produced is defective, and only a few infected cells have an intact open reading frame to produce infectious virions to infect another cell [27–29]. Thus, despite the many barriers required for the initial viral infection and spread, HIV successfully infects more individuals and remains in the infected individual’s body in viral reservoirs until death. Also, upon ART introduction, HIV adapted to multiple drug treatments as well as to several vaccine trials that failed due to the molecular adaptation of the virus and the diversity of viral reservoirs in different tissues. Currently, it is clear that HIV infection, acute and chronic, is highly dynamic and the evaluation of systemic replication is not a good representation of the constantly adapting viral reservoirs in different tissues. These research areas are novel and require extensive analysis to reach a cure.

**Figure 1:**
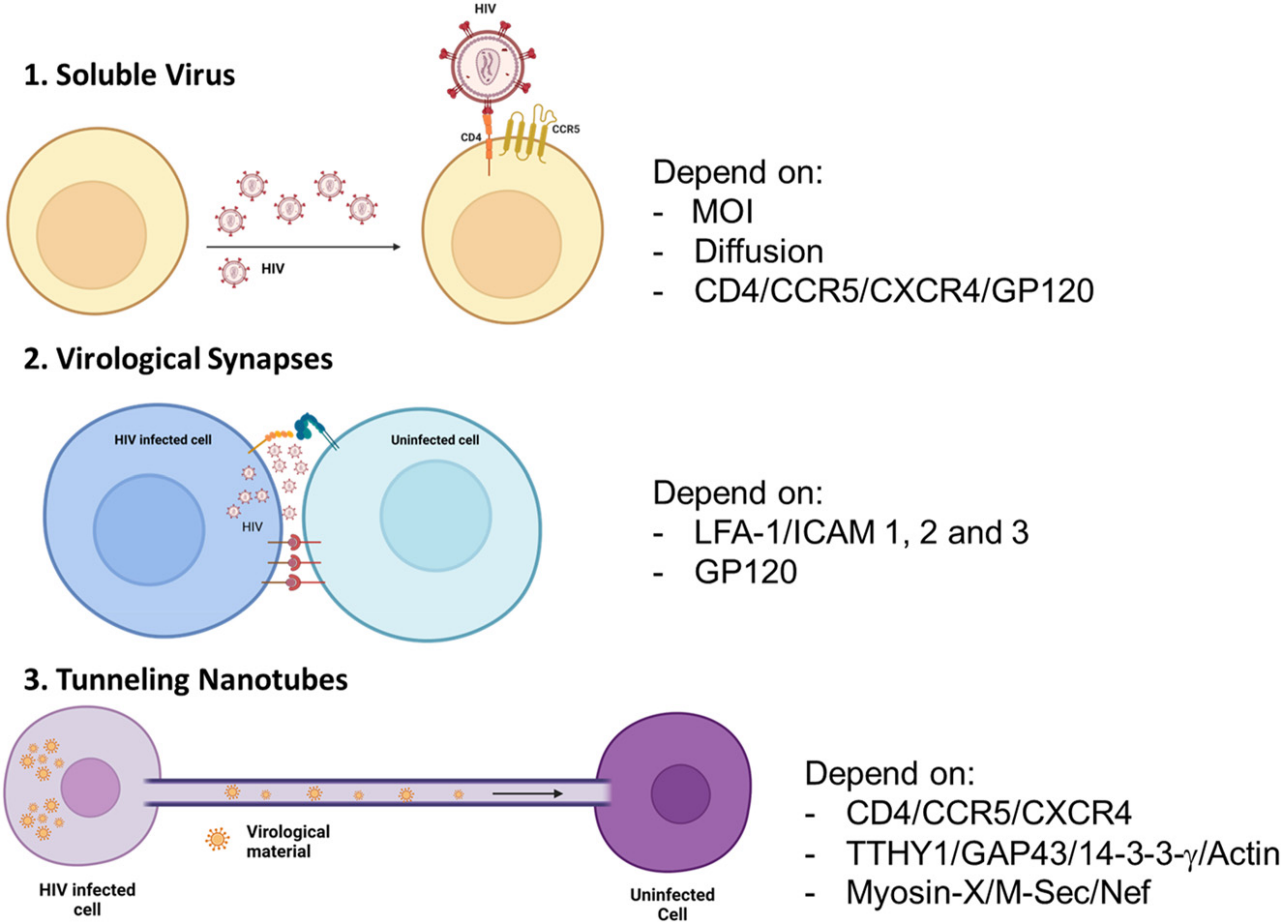
Models of HIV infection and reactivation. First, soluble or cell-free virus, depending on viral budding and diffusion into uninfected cells, MOI, viral diffusion, CD4/CCR5/CXCR4, and gp120 are essential for the success of this mechanism. Second, are the virological synapses (VS) that enable viral spread through direct cell contact and targeted budding. These mechanisms depend on LFA-1, ICAM-1, 3, and gp120. Third, tunneling nanotubes (TNTs), a long communication system depending on CD4, CCR5/CXCR4, actin, and nef, M-sec, Myosin-X, TTHY1, and 14-3-3-γ proteins.

It is well accepted that soluble or cell-free virus in the circulation is a major indicator of systemic infection and reactivation. However, the mechanisms of viral spread during the early stages of infection and reactivation, when the levels of the soluble virus are low to undetectable, are unknown. Most groups assume that successful viral infection and reactivation under ART depends on the random secretion of the soluble cell-free virus to reach uninfected cells (Figure 1, soluble virus). However, *in vitro*, *in vivo*, and *in silico* experiments shows that it is difficult to reconcile these ideas of random soluble virion infection with a highly effective infection and spread due to the multiple host and viral restrictions described above. To address this knowledge gap, several groups identified that virological synapses, VS, could provide an alternative mechanism of targeted infection not dependent on viral diffusion (Figure 1, virological synapses). VS are specialized pathogen-induced cellular structures that facilitate the cell-to-cell transfer of HIV and HTLV-1 [30–35]. Interestingly, the formation of VS increased HIV replication compared to soluble viruses up to 10,000-fold [36–38]. Many groups propose that cell-to-cell infection enables the virus to increase infectivity and viral fitness by avoiding immune surveillance, neutralizing antibodies, and promoting ART resistance [39]. However, the VS formation dependent on close cell-to-cell contact and HIV infection restricts cellular movement [13, 40, 41]; in consequence, reducing the potential interactions between HIV and uninfected cells [42, 43]. Currently, it is well-accepted that HIV is transmitted by two main mechanisms, soluble virus and the VS [44]. VS require the assembly of infectious virus particles [45–47], like soluble virus, which are released into a confined space between 2 or more cells. The VS shortens the generation of viruses by 0.9 times and increases viral fitness by 3.9 times [48]. However, even the best mathematical models cannot reconcile the patient data with the virological/immune/HIV cell cycle data [39, 49], [50], [51], [52], [53]. Thus, we propose that part of this missing link is tunneling nanotubes (TNTs) induced during the early stages of infection and reactivation (Figure 1, Tunneling nanotubes).

**Figure 2:**
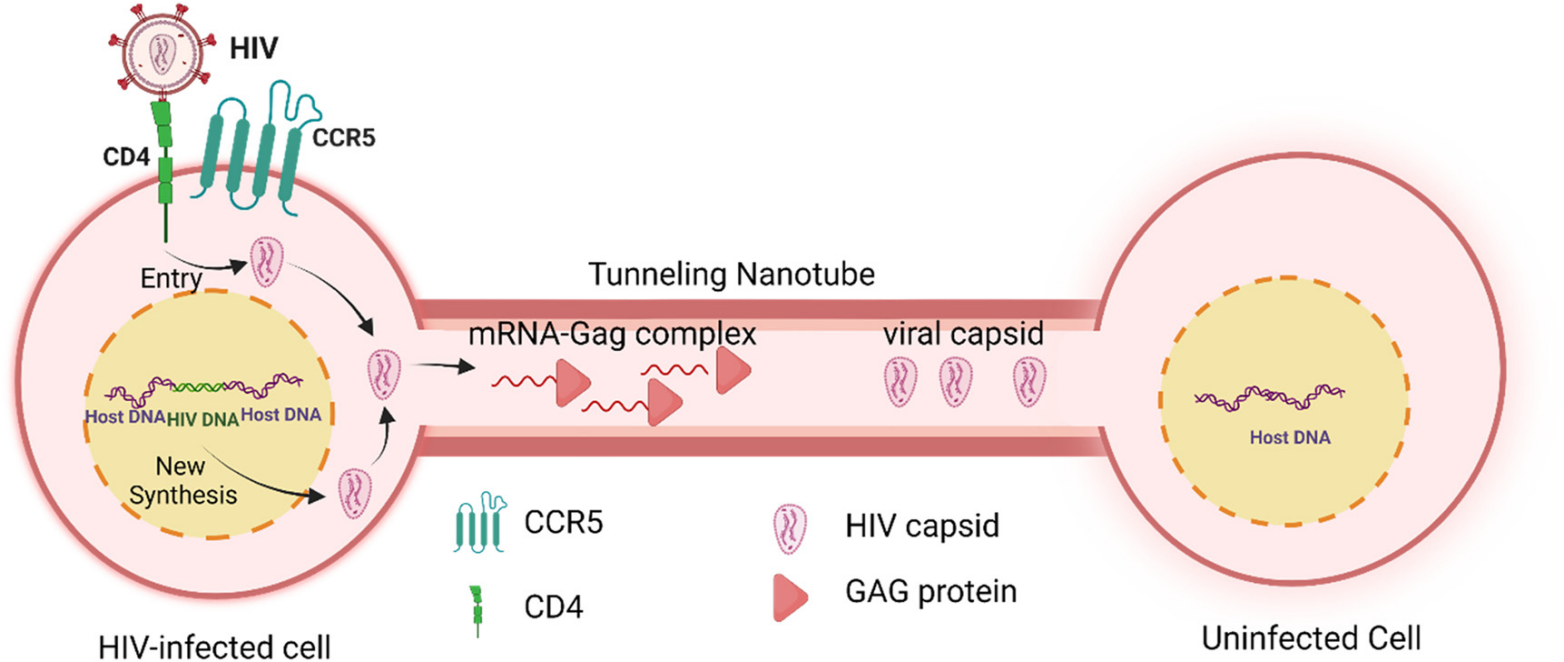
Model of long-range HIV infection and spread mediated by TNTs. TNTs are induced by acute HIV infection or reactivation, even in the absence of soluble virus or VS. TNTs could target uninfected cells up to 500 μm from the site of infection. This cartoon showed that upon HIV infection, TNTs allow the spread of a gag-mRNA complex or viral capsid into the uninfected cell.

Overall, as indicated above, the mechanism of HIV infection, viral spread and reactivation can look inefficient if dependent only on soluble virus or HIV-infected cells movement within the tissues; however, the virus and incoming HIV-infected cells had specific survival mechanisms to assure proper viral spread. The viral life cycle is well known in a single cell, and these data provided the basis for the discovery of antiretroviral drugs. However, these data do not explain the highly efficient early infection and reactivation steps under chronic ART conditions. Currently, several variables are poorly considered in cure strategies and reactivation, including viral latently and reactivation rates, viral reservoir variety and tissue specificity, and the critical point of cell-to-cell spread and localized inflammation. Several groups have estimated that one infected cell can cause a resurgence and produce 10^3^–10^4^ virions [22, 24]. In lymphoid tissue, studies have shown 10^8^ CD4^+^ T-lymphocytes produced infected with a steady-state residual viral release of ∼500 virions to maintain low to undetectable levels of systemic replication [54, 55]. However, upon ART interruption, a rapid rebound of the virus occurs [9, 11], [12], [13], [14]. In addition, as described above, the released viruses are subjected to stability issues and associated toxicity (Figure 2). Thus, the repopulation of the entire body with the virus to detectable systemic levels requires specific mechanisms of viral spread that cannot be explained by the local overproduction of soluble virus and its random diffusion into cells that can replicate the virus, especially considering the extremely limited number of viral reservoirs upon long-term ART. We propose that some of these amplification mechanisms are mediated by Tunneling Nanotubes (TNTs).

TNT differs from filopodium, first, by the length and the cellular origin of the cellular processes as well as the mechanisms of induction, TNTs in adulthood are only present in disease conditions. Filopodia are ∼2.37–5.8 μm, but TNTs can reach *in vivo* and *in vitro* distances of 500 μm. Both processes’ diameters are between 50 and 200 nm [56–58]. Second, TNT structures establish a bridge between the cytoplasm of connected cells, while filopodia do not, enabling the transfer of cargo and signaling molecules between connected cells. TNTs’ major component is F-actin, which are transient structures that can polymerize and depolymerize in 30–60 s and remain stable for several minutes to hours to significant and functional cytoplasmic exchange [57, 59]. It has been proposed that TNTs formation involves several proteins, such as M-Sec [60], LST1 [61], and Myo10 [62]. TNTs are an active communication system that enables “sick/infected” cells to establish long-range cytoplasmatic bridges to sensitize and spread bacteria and viruses between connected cells [63–70]. Our laboratory is one of the few working on HIV transmission *in vitro* and *in vivo* to answer the above questions during NeuroHIV, early stages of infection, and reactivation [64, 67, 71], [72], [73], [74], [75]. TNTs can extend up to 500 µm *in vitro* and *in vivo* [57, 58, 63], [64], [65, 67, 72, 76], [77], [78], [79], [80], [81], [82], [83], [84], [85], [86]. Also, TNTs are induced by acute and reactivating viruses; however, upon significant infection of neighboring uninfected cells (70% of the cells), TNTs disappear [87]. These data indicate that HIV only induces and uses TNTs during critical periods of HIV amplification and spread events. TNT formation from viral reservoirs upon reactivation is highly specific; most TNT are generated from latently infected cells into uninfected cells that could support viral replication. TNTs are not formed with uninfected cells that could not support viral replication, enhancing the virus capacity to target susceptible cells and assure successful infection and replication, even without significant soluble virus or formation of VS. TNTs share several mechanisms described for the VS-mediated HIV transmission, such as the requirement of adhesion molecules, and accumulation of viral proteins at the tip of the TNTs. TNTs mediated infection provides viral protection against immune recognition and neutralizing antibodies. In contrast, TNT is also unique due to the long-distance mediated communication, cargo transporters including organelles, and viral components, the lack of evidence of local viral assembling at the tip of TNTs, and the selectivity of some TNTs to specific viral products such as viral mRNA and viral proteins. However, the mechanisms of formation, stability and related transport remain unknown, and we hope that more laboratories become interested in this exciting area of research.

## Discovery, definition, and function of tunneling nanotubes

In 2004, Dr. Hans-Hermann Gerdes described thin nanotubular structures connecting cells by long range processes that allowed the intracellular transfer of vesicles and organelles, named TNTs [88, 89]. The first description of TNTs *in vitro* was made in rat kidney cells (PC12 cells), human embryonic kidney (HEK), and normal rat kidney (NRK) cells by three-dimensional (3D) live-cell microscopy [88]. TNT formation has been described in dendritic cells [90], mast cells [91, 92], cancer cells [93–96], neuronal/glial cells [78], and immune cells, including B cells, T-cells, NK cells, neutrophils, monocytes [64, 67, 74, 78, 87]. *In vivo*, TNT-like structure formation has been observed between immune cells in lymph nodes [78, 97], [98], [99], Drosophila [99, 100], and gut [101, 102]. TNTs have been reported in cancer [71, 95, 103], including glioblastoma [71, 95, 104], [105], [106] and pancreatic cancer [79, 81, 107], neurodegenerative diseases, including Alzheimer’s and Parkinson’s disease [64, 108], [109], [110], [111], viral infections including HIV [65, 67, 72, 73, 87, 112, 113], herpes virus [69], influenza A [114], and SARS-CoV-2 [59, 115] and bacterial infections including tuberculosis [44, 63]. All diseases that require efficient amplification or transport of aggregated proteins or infectious agents into neighboring healthy cells or tissues [86, 115, 116]. Additionally, oxidative stress and chemotherapy drugs increase TNT formation [94, 95, 117], suggesting that chemotherapy-induced stress could help some tumor cells to adapt to the pathogenic environment. However, further investigation is needed into the mechanism by which TNT formation is induced by disease conditions and the cargo transmitted between communicated cells.

TNTs have also been called tumor microtubes, cytonemes or filopodial bridges [99, 100, 118, 119]. TNTs establish a tubular conduit between connected cells to favor the directed exchange of small signaling molecules and cytoplasmatic content, including large organelles such as mitochondria and different kinds of vesicles [80, 118]. TNTs are uniformly F-actin positive and have low tubulin expression [76, 83, 84, 120], [121], [122], [123], suggesting that actin regulators and action-driven motors may be implicated in the formation and associated transport mediated by TNTs. F-actin mild blockers, such as Latrunculin or Jasplakinolid [113, 124], or F-actin depolymerization drugs, such as F-actin depolymerization cytochalasin B, inhibit or prevent TNT formation [125]. Due to toxicity, none of these unspecific treatments could be used in clinical settings. However, several groups in cancer and the HIV fields recently identified developmental proteins such as TTHY1, Connexin43 (Cx43) and GAP43 that could be used to prevent TNT formation and their role in spreading disease [103, 106, 126].

At least two different TNTs types have been described: (1) open-ended TNTs capable of direct cell-to-cell communication to exchange large cargo and organelles (discussed below) and (2) TNTs with a synaptic connection to adhesion molecules at the tip of the TNT. The synaptic kind of TNTs is mediated by long-range gap junctional communication [73, 126, 127]. In particular, Cx43, a gap junction protein, is present at the tip of the TNTs in close contact with the targeted cell [73, 103, 126]. Cx43 C-terminal tail binds to tubulin, and the full-length Cx43, sediments with microtubules, inducing filopodia formation [128, 129]. Also, Cx43 may play a key role in TNT formation that is regulated by gap junctional communication [57]. It has been demonstrated that TNT-communicated cells connected to more than two cells show a stronger electrical coupling than those connected by one TNT, suggesting that gap junctional communication correlates with better TNT-mediated communication [130]. Also, Cx43 expression enables long-range propagation of calcium (Ca^+2^) signals between TNT-connected cells [131]. Also, Cx43 knockdown correlated with a reduced number of TNTs and cargo transferred between connected cells [103, 129]. Further, we identified that Cx43 at the tip of the TNT between HIV-infected and uninfected macrophages is essential at initial events of infection, viral colonization, and HIV reactivation [73]. Blocking Cx43 with the gap junction blocker 18-alpha-glycyrrhetinic acid (AGA) or bocking mimetic peptide shows no significant differences in TNT formation but abolishes the inter-cellular signaling and transfer of organelles between TNT-communicated cells [73]. But we also propose that TNT-mediated communication in HIV reservoirs with uninfected surrounding cells could exchange viral components. In agreement, publications in the glioblastoma field also identified Cx43 as a key protein for tumor formation, spread, and associated inflammation [103, 132].

The most characteristic TNT property, in addition to the electrical coupling or communication, is their ability to transfer cytoplasmic cargo, such as organelles (mitochondria, endoplasmic reticulum, and vesicles) [133], lipids and liposomes, RNA, enzymes, proteins, transcription factors, and viruses at long distances between the connected cells [134]. In the HIV context, the transfer of material by TNT is extremely selective to help to spread infection, promote the immune response, regulate gene expression, and promote survival [67, 72, 73, 87]. TNTs transfer functional mitochondria into the targeted cells changing the recipient cell’s metabolic profile or other functional properties of the TNT-targeted cell [73]. As demonstrated in cancer, it has been proposed that Myosin-Va can facilitate the transport of the cargo inside of the TNT [88, 135], in particular mitochondria, through the interaction between these organelles and the mitochondrial Rho small GTPase (MIRO) [136]. In glioblastoma, TNTs mediated a selective and efficient transfer of compromised mitochondria with specific mutations in the mitochondrial DNA that change the metabolism of the TNT-targeted cells [137–139]. Also, the DNA repair enzyme O^6^-methylguanine-DNA methyltransferase (MGMT) is transported via TNTs from Temozolomide (TMZ)/irradiation (IR)-resistant cells to tumor-sensitive cells, likely to prevent cell death [95]. These data indicate that HIV and glioblastoma induce TNTs in a cell stress-dependent condition (HIV infection and reactivation or GBM treatment, TMZ and radiation) and result in the rapid viral and metabolic transfer to spread HIV infection and protect neighboring tumor or healthy cells from treatment. Both are essential components to reaching a cure for HIV and glioblastoma.

Nevertheless, TNTs are sensitive to several conditions, including light excitation and mechanical stress, leading to rupture [140, 141]. Radiofrequency (RF) has also been reported to retract TNTs after 4 h of exposure in AsPc-1 and PANC-1 cells. However, RF after 5 h resulted in increased expression of TNTs due to stress. Also, TNTs can transfer RNA between communicated cells, altering the targeted cell’s function, suggesting that TNT transfer is highly efficient [142, 143]. We propose that viral transfer via TNTs may help viruses to amplify infection and evade the immune response [67, 72, 73]. The mechanism of TNT formation and associated function is discussed below.

## Mechanism of TNT formation

There have been several proposed mechanisms by which cells can regulate TNT formation. One proposed mechanism involves Myosin-X [62, 82, 122], a motor protein associated with filopodia formation and dynamics [144, 145]. Myosin-X isoforms play an important role in TNT development in neuronal cells [122]. Myosin-X tail structure possesses the myosin tail homology 4 (MyTH4) domain, involved in microtubule binding [146] and can interact with other proteins associated with cytoskeleton development to regulate TNT formation and associated cell-to-cell transport [147, 148]. Inhibition of the MyTH4 domain inhibited TNT formation and spreading; in contrast, overexpression of the full-length Myosin-X increased TNT formation, suggesting that Myosin-X uses MyTH4 to interact with microtubules to aid in the formation of the TNTs [144, 147]. Furthermore, in osteoclasts, immunolocalization of Myosin-X is associated with the outer edges of immature TNTs and showed an interaction with β-tubulin at this early stage, suggesting that Myosin-X may act as a linker between the microfilament and the microtubule arm [144]. NF-κB ligand (RANKL) stimulation induces the formation of TNTs in osteoclasts accompanied by an induction of the expression of the *m-sec* gene and Myosin-X increase [145]. However, *m-sec* gene expression was not significantly changed when Myosin-X expression was reduced in osteoclasts, suggesting that Myosin-X regulates TNT formation independent of *m-sec* expression [147]. Additionally, if myosin-X is overexpressed, TNT formation occurs during the early stages of osteoclast differentiation by a mechanism mediated by SMAD 1/5/8 activation and NFATc1 expression and/or localization [147]. Additional studies must be done to understand Myosin-X-regulated TNT formation mechanisms.

Environmental factors could promote the formation of TNTs in cancer cells, including inflammatory mediators, such as cytokines, present in a conditioned medium from macrophages [149] or astrocytes treated with LPS plus IFNγ [72, 150]. Our group in 2003 published a controversial manuscript showing that human macrophages and monocytes generate gap junctional communication by TNF-α and IFN-γ [150]. In this manuscript, for the first time, we demonstrated that gap junctional communication can reach long distances by TNT-like structures contributing to monocyte transmigration and secretion of MMPs [150]. Later, the leukocyte-specific transcript 1 (LST1), a small protein expressed under inflammatory conditions [151], has been shown to regulate the generation of TNTs [152]. In HeLa cells, overexpression of LST1 induces the formation of TNTs by promoting LST1 interaction with myosin II, RalA, M-Sec, and filamin [61, 153]. Cellular oxidative stress can induce TNTs [94, 96, 133, 154], as was shown in astrocytes and cancer cells treated with H_2_O_2_, by activating the mitogen-activated protein kinase (p38 MAPK) pathway [154]. Studies in hippocampal astrocytes and neurons treated with H_2_O_2_ showed more TNTs. The TNT formation was directed from the stressed to the unstressed cells in co-cultured cells, suggesting that TNT formation is a defense response to cellular stress [133]. However, whether the nature of the H_2_O_2_-induced TNTs is similar to TNT induced by viruses or bacteria is unknown. Similar results were shown in neurons and astrocytes that use TNTs to transfer mitochondria between cells as protection to prevent apoptosis in response to several conditions, including stroke [117]. It has been reported that the transcription factor p53 regulates actin cytoskeleton remodeling in metastasis, and cell invasion [155]. H_2_O_2_, induce the activation of p53, epidermal growth factor receptor, and the Akt/PI_3_K/mTOR pathway as a cellular response to stress, suggesting that TNT development may have an important role in preventing cancer formation and neurodegeneration [133]. Furthermore, TNT induced by H_2_O_2_ and serum deprivation can also result in the overexpression of M-Sec in wild-type astrocytes but not in p53-deficient cells, suggesting that M-Sec might be regulated by p53 activation [133]. M-Sec is a membrane protein and, when interacting with Ral GTPase, can induce the formation of TNTs, remodeling the actin cytoskeleton and vesicle trafficking, as shown in a macrophage cell line [156]. These findings indicate that M-Sec, p53 and associated pathways play an important role in TNT formation induced by cellular stress and may represent a promising therapeutic target to prevent TNT formation.

## Tunneling nanotubes in HIV

The current agreement is that most HIV-infection occurs by cell-free virus [157–160] and close cell-to-cell transmission by VS [33, 158, 161], [162], [163], [164]. However, in conditions with few HIV-infected cells (acute infection and latently infected viral reservoirs) and minimal to undetectable systemic viral replication, alternative mechanisms of directed infection and viral spread may be used for the virus. Several groups propose that the VS enables close cell-to-cell infection, where the virus is released into a small intercellular area between an infected and an uninfected cell to enable targeted HIV infection [165–167]. We propose that TNTs could be an additional mechanism of long range HIV targeted infection and reactivation.

The first description of TNTs in the context of HIV was in primary human macrophages, showing that HIV infection increases the number of TNTs three days post-infection [67]. Surprisingly, TNT formation and communication are highly selective because the HIV-infected cells targeted only uninfected cells at distances up to 500 µm [67]. Eight days post-infection, when HIV infection reached more than 80% of the cells, the number of TNTs was reduced, suggesting that TNTs are only induced during the early steps of viral reactivation and spread [67, 73, 87]. In agreement, data obtained using co-infected macrophages with *Tuberculosis Bacillus* and HIV indicate an increase in TNT formation during the process of reactivation elicited by the bacterium [44]. Blocking TNTs prevented HIV cell-to-cell transfer and overproduction in M(IL-10) macrophages [44].

*In vivo*, TNTs are expressed in tissues from NOD/SCID IL-2 RG−/−(NSG) humanized mice infected with HIV_ADA_ or with low numbers of HIV_ADA_-infected leukocytes [87]. After the initial viral entry mediated by CD4 and CCR5/CXCR4, the treatment with a CCR5 blocker (TAK779) did not affect tissue seeding or spreading of the virus, despite observing reduced systemic viral replication [87]. Thus, TNTs are induced by HIV infection and communicate HIV components between human HIV-infected and uninfected human cells [73, 87, 113]. These data were the first demonstration that HIV-infected cells can target surrounding uninfected cells and transfer viral material between TNT communicated cells *in vivo*. We demonstrated that TNTs are highly selective to identify and only form TNTs with cells that can support active replication [87], suggesting an exquisite cell selectivity even in In vivo situations. Our data in an HIV mouse model tested with TAK779 indicated that in tissues, TNTs are increased in response to reduced systemic viral replication due to high levels of TAK779 [87]. In these experiments, we identified at least three kinds of viral behavior depending of the tissue analyzed and probably according to the accessibility to the bloodstream, brain, lymph nodes, and liver/spleen [87]. Brain>lymph nodes>liver/spleen>and blood had a different TNT formation and viral seeding, both independent of the systemic replication. A concerning piece of data in these animals is that some classes of ART, despite reducing systemic viral replication, enhanced viral spread and numbers of viral reservoirs in a tissue-type dependent manner. Overall, we propose that TNTs *in vitro* and *in vivo* enhance HIV spread and probably help to “protect” viral reservoirs in different tissue compartments as well as contribute to viral adaptation and diversity.

To examine the nature of the material transferred by TNTs between HIV-infected and uninfected cells, we performed transmission electron microscopy of sagittal TNT sections formed between HIV-infected and uninfected macrophages. We could not detect HIV-like core structures inside or along the TNT [73]. However, by confocal, the viral RNA, gag, reverse transcriptase (RT), and integrase were detected inside of the TNT and actively communicated from the HIV infected into the uninfected cells. Further, the microinjection of the entire virion into primary cells induced to express TNT by H_2_O_2_ treatment indicated that TNTs are impermeable to the entire virion. However, microinjection of the viral RNA or viral proteins, except gp120, were actively transferred between communicated cells. A similar mechanism was described in the model where macrophages infected with HIV form TNTs with adjacent B cells (generally not infected with HIV) and transfer only the Nef protein. The presence of Nef in B cells inhibits the immune response systemically and at the mucosal entry sites [168]. Also, these data are relevant in the current ART era because we detected residual viral replication in viral reservoirs and bystander transfer into neighboring uninfected cells, compromising their function [75, 169, 170]. Coculture of reactivated infected cells with fibroblast, epithelial cells, mouse cells, and human uninfected macrophages only resulted in TNT formation between the infected cells and the human susceptible cells, human macrophages, suggesting a highly selective mechanism of TNT formation and associated transport [67, 72, 73]. Thus, we propose that HIV uses TNT communication to spread the infection and prevent an effective immune response. However, the nature of the cargo and transferred material and whether it could be infectious are still under active investigation.

As discussed, HIV induces TNT formation, but the mechanism is poorly understood. Previous studies have shown that Nef’s protein-negative factor could play a key role in TNT formation [171]. Our observation validated prior studies, showing that Nef is transferred by TNT-connected cells in macrophages via a myosin-X-dependent mechanism [62]. Nef protein promotes viral spread and disease progression *in vivo,* and it has been proposed that this protein in conjunction with M-sec promotes TNT formation [112]. In addition, proteomics analysis showed that Nef is associated with proteins that form the exocytic complex identified within TNTs. Moreover, exocyst complex 2 (EXOC2) was reported to have enhanced TNT formation under HIV [171]. According to these findings, macrophages require M-sec and Ral/exocyst complex to form TNT communication [61, 112, 156]. Understanding these interactions between the viral proteins and the exocyst complex will help us discover a therapeutic strategy to block TNTs under HIV infection. A similar mechanism has been described in SARS-CoV-2 and influenza, where TNTs contribute to immune evasion and the spread of infection [59, 68, 114, 115, 172], [173], [174].

## HIV brain viral reservoirs and NeuroHIV

The development of ART has changed the life expectancy of HIV-infected individuals, particularly in developed countries where ART is accessible. But long-term ART results in several complications associated with drug toxicity [175–177], drug-drug interactions [178, 179], adherence [180], stigma [181], and associated-cost [182]. In addition, aging-related diseases have become the main issue in the current infected population, including NeuroHIV. A key feature of NeuroHIV is the early invasion and infection of the brain, including the early formation of long-lasting HIV brain reservoirs. A viral reservoir is a long-lasting HIV-infected cell where the virus remains silent, and upon reactivation, the dormant virus is reactivated [8, 170, 183]. Upon the introduction of ART, the hope was that therapy would reduce the size of the viral reservoir pool. However, calculations in T cells indicate that the reduction of the pool is too slow to reach a functional cure [184]. Recently, our laboratory, in collaboration with the NIH AIDS repository, the National NeuroAIDS tissue consortium (NNTC) center, and the Neurobiobank, identified, quantified and characterize HIV reservoirs within the brain of individuals that have undergone 0–23 years of ART by *in situ* hybridization [169]. We identified that in brains obtained from HIV-infected individuals, microglia/macrophages are the main cell type infected with HIV; however, a small population of astrocytes is also infected. The first two years of ART strongly decreased the number of infected cells; however, the viral reservoir pool remained constant for up to 12–14 years on ART. After 14 years, the pool of microglia/macrophages decreased, but astrocytes were not affected by ART, suggesting that some viral reservoirs are not susceptible to long-term ART [169]. More important for this manuscript, we observed bystander residual secretion of several HIV proteins into neighboring uninfected cells. However, the viral protein secretion was not random, and we believe that TNTs may be involved in these toxicity mechanisms. Also, we propose that TNTs play a key role in bystander damage in ART conditions [63].

The most described viral reservoir corresponds to resting CD4^+^ T lymphocytes due to easy access to blood products [185–187]. Besides the viral reservoir in the peripheral blood, viral reservoirs have been described in tissues such as lymphoid tissues, intestinal tissues, lungs, skin, and central nervous system, even in patients under ART [169, 186, 188, 189]. The mechanism of viral silencing has been reported to be at the transcriptional level; however, the silencing mechanisms are still under active investigation [190].

It has been demonstrated that HIV-1 transcription depends on a varied and complex interaction of host cell transcription factors with the viral long terminal repeat (LTR). The LTR of the virus includes DNA regulatory elements, including the promoter comprised of SP1 binding sites, a TATA element, and a highly active initiator sequence [191]. Also, the HIV LTR has an enhancer sequence that contains two tandem NF-kB motifs that can bind NF-kB and other binding members [192]. Several groups demonstrated that this enhancer is essential for viral reactivation [192]. HIV transcription is also regulated by the transcription initiation complex and elongation. Currently, there are no specific protein complexes associated with transcriptional repression or latency; instead, most latency and reactivation have been associated with the expression of the transactivator of the virus, HIV-Tat [193, 194]. Viral reactivation in the absence of HIV-Tat can be initiated, but only short abortive transcripts are produced. HIV-Tat’s main function is to orchestrate the cellular transcriptional elongation factor p-TEFb to the nascent RNA polymerases by binding to the HIV TAR element, an RNA stem-loop 3D structure found at the 5′ end of viral transcripts. P-TEFb, a protein kinase complex comprised of Cyclin T1 and CDK-9, stimulates HIV elongation by removing anti-elongation factors.

In viral reservoirs, the sequestration or inactivation of P-TEFb is a strong signal for viral reactivation of latent proviruses [10, 191, 195, 196]. Further, NF-kB and NFAT are sequestered in viral reservoirs in the cytoplasm [192, 197], [198], [199], [200]. However, NF-kB and NFAT activated upon TCR stimulation move into the nucleus of a PKC and are calcineurin-dependent. Further, NF-kB and NFAT binding to the HIV elements induces viral reactivation by the direct recruitment of histone acetyltransferases to the LTR [198] to acetylate histones and provide the signals for the recruitment of the chromatin remodeling complex BAF by displacing the restrictive nucleosome-1, a sequence positioned immediately downstream from the transcriptional start site. Upon infection, HIV transcription is silenced by epigenetic-related mechanisms. Several mechanisms have been proposed to reactivate the virus to kill the cells with active replication, T activators, PKC activators, and histone deacetylase [201–205]. However, the transcriptional silencing and reactivation mechanism are complex, and one mechanism cannot account for all viruses or cell types with integrated viral DNA. Further, it is unknown whether any of these mechanisms are associated with TNT formation and transport, probably because similar pathways will be activated during the early stages of infection and reactivation, which induce replication and promote TNT formation.

Different strategies have been proposed to eradicate viral reservoirs to enhance the immune response against infection using controlled *in vivo* exposure to HIV-1 antigens [206]. Theoretically, these vaccines could provide antigen-specificities and functionality of anti-HIV T-cell responses to eliminate infected cells and facilitate long-term viral control in the absence of ART [207]. The “shock and kill” strategy has been proposed as an alternative approach to achieving the HIV cure. It involves the use of latency-reversing agents (LRAs) to induce the transcription of the virus in the infected cells (“shock” step), leading to HIV RNA synthesis, viral protein production, and the release of the virus. Sequentially, the infected cell is killed by the acute/toxic infection or by the patient immune system (“kill” step) [208, 209]. However, a significant reduction in viral reservoirs has not been achieved to reach a cure. The use of neutralizing antibodies to target specific sites on the HIV envelope could further enhance viral reservoir elimination by enhancing immune recognition, however, viral reservoirs had mechanisms of immune evasion inlcuding TNTs [210]. Another alternative to eliminate viral reservoirs is “block and lock” therapy, with the major goal being to permanently silence all proviruses, even after treatment interruption [211]. Also, gene-editing tools such as CRISPR/Cas9 appear as an option to eradicate viral reservoirs by targeting the HIV-proviral DNA in the latent reservoir with the goal of either excising the provirus or portions of the provirus with two simultaneous CRISPR/Cas9 cleavage events or ablating viral promoters to silence transcription [212–214]. All these strategies are under evaluation, and so far, they are not able to efficiently kill the viral reservoirs. We propose that blocking TNTs results in the accumulation of viral proteins by preventing their diffusion into surrounding uninfected cells enhancing viral protein toxicity and immune recognition of viral reservoirs.

As indicated above, CNS viral reservoirs remain despite long-term ART, and TNT helps them to survive. Our data indicate that TNTs, in addition to communicating viral components, also participate in promote differentiation of the TNT targeted cell by transferring mitochondria with specific mutations to change the metabolism of these cells into a glutamine/glutamate-dependent mechanism. Thus, long-lasting viral reservoirs could in the brain survive in areas with abundant amino acids or neurotransmitters [9, 170]. This metabolic profile has been observed in glioblastoma stem cells to survive the tumor microenvironment [215–217]. More importantly, the metabolic adaptation is mediated by TNTs that enable the metabolic shift in viral reservoirs. Blocking TNTs or these pathways used by viral reservoirs results in significant apoptosis and short-term survival of latently infected cells [9, 170]. We believe that TNT communication enables the sharing of mitochondria that had metabolic compromise but cannot also trigger apoptosis. We identified that surviving macrophage/microglia prevent apoptosome formation due to high BIM expression [170]. These data differ from those described in T cells, where BCL-2 is a major player in the survival of lymphocytic viral reservoirs [218, 219]. We did not observe changes in the expression or localization of BCL-2 in active and latent infected macrophages or microglia as described in T cells [219]. We identified an incomplete formation of the transition pore on the surface of the mitochondria, enabling the “leak” of cytochrome C into the cytoplasm to trigger apoptosis but not enough to induce a proper formation of the apoptosome [9, 220]. All these components are shared by TNTs and adapt the myeloid viral reservoir for extended survival.

A similar mechanism was described in human astrocytes *in vivo* and *in vitro*. Astrocytes are the more abundant cell in the nervous system, and experiments *in vivo* and *in vitro* demonstrated poor infectivity and replication of astrocytes [75, 221, 222]. However, despite the low ratio of infected astrocytes, the overall number is high due to the abundance of this cell type in the brain. *In vivo* models showed that less than 3% of astrocytes in the brain can become viral reservoirs [75, 222]. Our laboratory examines astrocytes as viral reservoirs, including their characterization and role in the current ART era. We developed a long-term model system to examine viral silencing in human primary astrocytes. Long-term infection of astrocytes resulted in spontaneous viral silencing even in the absence of ART, suggesting that astrocytes have intrinsic mechanisms of viral silencing [75]. This provides unique opportunities to examine viral silencing and reaction upon treatment with specific reactivators. An *in vitro* model for latency showed that cell cultures of primary astrocytes, macrophages, and microglia after 120 days post-HIV infection become silent and do not show HIV p24 production [75]. After 120 days post-infection, with the addition of LRAs, the reactivation of the virus was transient, and HIV replication became silent again [9, 75]. However, viral transfer into T cells, macrophages, and several cell lines required TNT-like structures. The cell-free virus could not transfer the virus from astrocytes into other cell types.

Our data from human glioblastoma cancer cell lines, as well as primary cells, showed that TNTs are rich in GAP43 and 14-3-3-γ protein and that upon chemotherapy treatment and radiation, tumor cells induce the formation of TNTs with neighboring non-tumor and tumor cells. We determined that TNT formation enables the exchange of a critical DNA repair enzyme, MGMT, from tumor cells insensitive to TMZ to sensitive cells. We believe that this targeted TNT-mediated communication and transfer of MGMT protects the tumor and surrounding cells from apoptosis induced by radiation and TMZ treatment [95]. A concerning issue in glioblastoma is that tumor cell IR or TMZ treatment induces TNT formation. We hypothesize that this cancer treatment-related induction of TNTs could “prime” tumor cells for adaptation [95]. However, in the context of HIV, several ARTs induce the formation of TNTs, suggesting potential adverse effects of the drugs in TNT formation and associated transfer.

In HIV, we propose a similar mechanism during acute infection and viral reactivation as described [75, 87]. In agreement with these ideas, our studies using a humanized animal model infected with HIV and treated with high concentrations of TAK-779 to prevent or reduce cell-free virus infection indicates that despite that TAK-779 treatment reduced systemic replication, TNTs proliferate in several tissues, including the spleen, lymph nodes, and brain [75, 87, 95, 221, 223], [224], [225]. We propose that TNTs formation in the absence of viral replication corresponds to an adaption of the viral tissue reservoirs to the decreased numbers of infected cells and replication. In tissues, we identified that TNTs transport viral proteins and RNA, suggesting a unique tissue adaptation to the changes in systemic viral replication; however, whether this tissue adaptation to other ART is still unknown and needs to be addressed for cure efforts.

Our data on HIV indicate that viral reservoirs have significant ER/mitochondrial/Golgi stress and compromise [221], resulting in the endoplasmic reticulum protein aggregation, including tau and APP, both essential components of aging-related diseases such as Alzheimer’s [226, 227]. TNTs could provide an alternative explanation for the high prevalence of Alzheimer-like diseases in HIV-infected populations and the transfer and diffusion of protein aggregates.

## TNTs as a potential mechanism for viral spread via TNTs during acute infection and reactivation

It is well accepted that soluble virus is a major indicator of systemic infection and reactivation. However, the mechanisms of viral spread during the early stages of infection and reactivation, where the soluble virus is low to undetectable, are unknown. Most groups assume that viral infection depends on budding and the random diffusion of infectious virions that need to reach a susceptible cell to infect them. Several groups identified that VS [44] could provide an alternative mechanism of infectivity mediated by cell-to-cell communication and dependent on adhesion molecules and localized budding. However, VS requires cell migration of susceptible uninfected cells into areas with viral reservoirs [39, 228, 229]. Thus, an alternative mechanism of infection and cell-to-cell spread must be considered. We propose that this missing link is the TNTs. Our data indicate that, First, we identified that viral reservoirs, upon reactivation, induce TNT formation and associated exchange of intracellular material, including host and viral components between communicated cells. Our data indicate that the material transmitted between viral reservoirs and uninfected cells promotes viral infection and spread [75]. Upon infection of most cells around viral reservoirs, TNTs are degraded, and soluble viruses become predominant, suggesting that TNTs are only required for initial viral spread. We propose that blocking TNTs, prevents effective viral reactivation, improves immune recognition, and enhances neutralizing antibody function to prevent subsequent infection; Second, TNTs are generated in viral reservoirs and reach only uninfected cells that can support viral infection and replication up to 500 µm *in vitro* and *in vivo* [67, 73, 75, 87, 137]. TNTs do not reach cells that do not support infection, making TNTs a unique, highly selective and efficient mechanism of targeted infection during reactivation; Third, TNTs serve as a intracellular path of infection insensitive to neutralizing antibodies and some types of ART; Fourth, we identified at least two different types of TNTs in primary immune cells, an open-ended that enables an infectious HIV component to be shared between HIV infected and uninfected cells, and a closed-ended TNTs similar than the VS but at long range [63, 73]; Fifth, we identified several unique TNTs biomarkers (mostly developmental proteins) that if they are blocked prevent TNT formation as well as HIV spread during acute and upon viral reactivation [75, 87]; Sixth, TNTs mediated HIV spread is in 10–100 folds more efficient than soluble virus during acute and reactivation events [87, 137]; Seventh, TNTs increased viral fitness and accounts for multiple viral copies integrated into a single cell as observed in HIV infected individuals [75, 87, 221]; Eighth, TNTs recognize susceptible uninfected immune cells at long distances and target them for infection increasing the efficient of infection in a Log scale [75, 87]; Nineth, most of our data were obtained in primary cells (macrophages and T-cells), relevant animal models (humanized mice/macaques), and patient samples (blood and tissues) [73, 75, 87, 169]; Tenth, high-resolution laser capture, subsequent proteomics and RNAseq analysis demonstrated that TNT mediates the transfer of infectious HIV [73], but also several developmental proteins; however, the nature of the transported cargo, and viral materials are unknown. Lastly, in macrophages, TB-induced IFN-gamma signaling induces cell surface expression of Siglec-1, which can capture virus particles via interaction with viral envelope lipids [66]; Siglec-1 expression may thus increase virus particles trafficked over the surface of TNTs. Therefore, how HIV-1 exploits TNTs for efficient virus spread may differ between TNTs formed under different conditions. All these mechanisms are novel, and we hope other groups become interested in exploring them to reach a cure.

## Mathematical modeling to viral spread: cell-to-cell transmission versus soluble virus infection

As discussed above, HIV can infect cells through soluble virus infection and cell-to-cell transmission, which are important during the acute infection and reactivation of the virus. In combination with experimental data, mathematical models have been used to evaluate the relative contributions from the two transmission modes. A static cell culture system allows viruses to perform both cell-free virus infection and cell-to-cell transmission, while a mildly shaking system can block cell-to-cell transmission, e.g., by preventing the formation of the VS between uninfected and infected cells [51]. Based on a basic viral dynamic model [24], Iwami et al. explicitly developed a model that included these two infection modes [230]. The model is given by a system of differential equations: d*T*/d*t* = *gT*[1−(*T* + *I*)/*T*_max_] − *βVT* − *ωTI*; d*I*/d*t* = *βVT* + *ωTI* − *δI*; d*V*/d*t* = *pI* − *cV*. Here the dynamics of target T cells (*T*) are modeled using a logistic term *gT*[1−(*T* + *I*)/*T*_max_] with the maximum generation rate *g* and the carrying capacity *T*_max_. Target cells are infected by either cell-free virus (*V*) at rate *βVT* or cell-to-cell transmission at the rate *ωTI*, leading to infected cells *I*. Infected cells are assumed to die at per capita rate *δ*. Virions are produced at a rate of p and cleared at a constant rate of *c*. Letting *ω*=0, i.e., no cell-to-cell transmission, they fit the model to the uninfected cell, infected cell and viral protein data collected from the shaking system and obtained parameter estimates for the cell-free virus infection [230]. Comparing the fitting and parameter values obtained by fitting the full model to the data collected from the static system, they estimated that cell-to-cell transmission accounts for approximately 60% of viral infection [230]. Thus, even complete inhibition of the cell-free virus infection would not be sufficient for viral control, and the inclusion of alternative mechanisms of infection and reactivation such as VS and TNTs needs to be consider for reaching a cure.

When cell-to-cell transmission occurs, and multiple virions are transferred from infected to uninfected cells, antiretroviral drugs may not inhibit the infection by all virions, leading to a higher probability of successful infection [231]. Thus, the cell-to-cell transmission may contribute to HIV persistence in patients under antiretroviral therapy. Mathematical models have also been developed to test whether cell-to-cell transmission can explain the low viral load persistence during suppressive therapy. Libin Rong and his collaborator divided the population of infected cells into different classes in a model by considering how many virions are transmitted during cell-to-cell transmission [53]. Given the experimental results in [231], it is reasonable to assume that the more virions transmitted during cell-to-cell transmission, the less effective the treatment at blocking the transmission. They found that the viral load is less sensitive to the drug’s effectiveness against cell-to-cell transmission, and thus, low viral load persistence is possible even when the treatment completely blocks cell-free virus infection [53].

HIV multiple infections occur far more frequently than single infection *in vivo* and *in vitro* [232]. It was found that CD4^+^ T lymphocytes in HIV-infected patients can harbor up to eight proviruses, with an average of three or four proviruses per cell [233]. Multiple infections were also observed in experiments *in vitro* [234–237]. Multiple infections containing diverse viral genomes are a prerequisite for the evolution of recombinant forms of HIV, resulting in viral escape from host immune responses and drug resistance to antiretroviral therapy [235]. Mathematical models have been used to investigate the mechanisms that underlie the high incidence of HIV multiple infections and to understand its implications on viral evolution and therapy [232, 238]. One explanation for multiple infections is cell-to-cell transmission. The other possibility is a sequential virus infection, i.e., each contact of CD4^+^ T cells with a cell-free virus results in the transmission of one viral genome to the target cell [232]. Rong and his collaborators developed and analyzed a mathematical model containing sequential cell-free virus infection and cell-to-cell transmission [239]. By comparing model prediction with the distribution data of proviral genomes in HIV-infected spleen cells [233], they found that multiple infections can be explained only when the two viral transmission modes are included [239]. Their model simulations suggest that most T-cell infections are attributed to cell-to-cell transmission, and this transmission mode accounts for more than half of a cell’s multiple infections [239]. Thus, cell-to-cell transmission is critical for multiple HIV infections. Now with the discovery of TNTs, these approaches needs to be re-evaluated, due to their highly specific mechanisms of targeted infection and viral spread.

HIV infection of other cells, such as macrophages, may also contribute to viral persistence during antiretroviral therapy. In addition to cell-free virus infection, macrophages can be infected when engulfing infected CD4^+^ T cells [240]. Rong and collaborators have used models to study the infection dynamics of CD4^+^ T cells and macrophages via cell-free virus infection and cell-to-cell viral transmission [241]. Parameter sensitivity and viral dynamics simulations show that even when the infection of CD4^+^ T cells is completely blocked by therapy, the virus can still persist, and the steady-state viral load is not sensitive to the change in treatment efficacy. The relative contributions to viral replication show that cell-free virus infection leads to most macrophage infection. Viral transmission from infected CD4^+^ T cells to macrophages during engulfment accounts for a small fraction of macrophage infection [241]. These modeling results suggest that several types of viral reservoirs can contribute to HIV persistence during suppressive therapy, and alternative mechanisms of infection and reactivation need to be examined to conciliate the biology and mathematical models, more importantly with the patient data.

## Conclusions

TNTs have become a potential target to prevent, revert, or block the transfer of infectious/damaging pathogens. Several groups identified biomarkers of TNTs, also named cytonemes or tumor microtubes, including Connexin43, GAP43, 14-3-3-γ, TTHY1, and several mitochondrial/vesicular markers [59, 95, 106]. Pathogens and pathogenic conditions “learn” to use TNTs to spread between cells, inside organs efficiently, and different body areas to perpetuate disease. More importantly, TNTs are not expressed in healthy individuals. The main issues in the field of TNTs are: (1) the unique nature of TNTs and their consideration as an efficient communication system used for several pathogens and pathologies; (2) the mechanism of TNT formation and associated transport are unknown; (3) how infected cells can detect and target TNT into uninfected or susceptible cells to target them; (4) the identification of potential blockers to prevent, revert, or block formation or associated TNT transport, and (5) the inclusion of TNTs into the knowledge of HIV cures and consider them essential for cure efforts due to their role in infection and reactivation. Our main goal with this review is to introduce to the HIV scientific community several issues not often discussed in the HIV field. The discovery of TNT and VSs in conjunction with the cell-free virus provides tools to explain the efficient mechanisms of HIV infection and reactivation.
